# Immediate and Persistent Effects of Temperature on Oxygen Consumption and Thermal Tolerance in Embryos and Larvae of the Baja California Chorus Frog, *Pseudacris hypochondriaca*

**DOI:** 10.3389/fphys.2019.00754

**Published:** 2019-06-18

**Authors:** Casey A. Mueller, Julie Bucsky, Lindsey Korito, Samantha Manzanares

**Affiliations:** Department of Biological Sciences, California State University San Marcos, San Marcos, CA, United States

**Keywords:** amphibian, carry-over effects, development, energy use, phenotypic plasticity, temperature

## Abstract

The developmental environment has significant immediate effects on phenotypes, but it may also persistently or permanently shape phenotypes across life history. This study examined how developmental temperature influenced embryonic and larval phenotypes of Baja California chorus frog (*Pseudacris hypochondriaca*), an abundant amphibian in southern California and northern Baja California. We collected egg clutches from native ponds in northern San Diego County within 24 h of fertilization, and clutches were separated and distributed between constant temperatures of 10, 15, 20, and 25°C for incubation. Oxygen consumption rate ($\dot{\text{V}}$O_2_), developmental stage, and embryo and yolk masses were measured throughout development. Time to 50% hatch, survival at 50% hatch, and hatch duration were determined. Development rate was strongly affected by temperature, with warmer temperatures reducing time to hatch and hatch duration. Survival to hatch was high across all temperatures, >90%. Mass-specific $\dot{\text{V}}$O_2_ of embryos either remained constant or increased throughout development, and by hatching energy demand was significantly increased at higher temperatures. There were limited temperature effects on growth, with embryo and yolk dry mass similar between temperatures throughout embryonic development. To examine long-term effects of embryonic temperature, we reared hatchlings from each temperature until onset of larval feeding. Once feeding, larvae were acclimated to 20 or 25°C (>2 weeks). Following acclimation to 20 or 25°C, we measured larval mass-specific $\dot{\text{V}}$O_2_ and critical thermal maximum (CTMax) at a common developmental stage (Gosner stages 32–36, “hindlimb toe differentiation”). Embryonic temperature had persistent effects on larval $\dot{\text{V}}$O_2_ and CTMax, with warmer temperatures generally resulting in similar or higher $\dot{\text{V}}$O_2_ and CTMax. This partially supported a “warmer is better” effect of embryonic incubation temperature. These results suggest that in a thermally robust amphibian species, temperature may program the phenotype during early development to construct traits in thermal tolerance and energy use that may persist. Overall, *P. hypochondriaca* displays a thermally robust phenotype, and it is possible that amphibians that possess a wider range of phenotypic plasticity will be relatively more successful mitigating effects of climate change.

## Introduction

The environment is a driving force that exerts immediate and long-term effects on phenotypes. However, animals are not passive entities, and phenotypic plasticity is an important avenue by which animals respond to the environment (Garland and Kelly, 2006; Mueller et al., 2015a). Phenotypic plasticity is a modification in phenotype to adjust and respond to the environment, occurring through changes in physiology, morphology and/or biochemistry (West-Eberhard, 1989; Pigliucci et al., 2006). Plasticity is particularly important for responses to temperature, which affects nearly all physiological processes. In particular, the embryonic and larval/juvenile period for vertebrates are typically very sensitive developmental periods in an organism’s life history, marked by fundamental shifts in size, morphology, and physiology (Mueller et al., 2015a,b; Smith et al., 2015). Responses to temperature during these stages may have significant implications for species success, particularly in response to increased temperatures predicted with climate change (Cayan et al., 2008; van Vliet et al., 2013; IPCC, 2014).

The effect of temperature in shaping developing phenotypes comprises a very important area of current comparative physiological research, particularly for amphibians, as higher global temperatures are contributing to unprecedented worldwide declines in many groups of amphibians (Stuart et al., 2004; Pounds et al., 2006; Cohen et al., 2018). Embryonic ectothermic vertebrates are often exposed to wide fluctuations in temperature, and a species’ ability to phenotypically respond to the environment may have profound consequences. Temperature drives development rate and growth of embryonic and larval amphibians and fish (Harkey and Semlitsch, 1988; Seymour et al., 1991; Mitchell and Seymour, 2000; Watkins, 2000; Mueller et al., 2011, 2015b; Smith et al., 2015), and can alter embryonic oxygen consumption rate ($\dot{\text{V}}$O_2_), heart rate, yolk-conversion efficiency (YCE), and hatching survival (Mueller et al., 2011, 2015b; Eme et al., 2015). For example, embryonic incubation at warmer temperatures results in persistently higher $\dot{\text{V}}$O_2_ values in embryonic and larval moss frogs (*Bryobatrachus nimbus*) and embryonic lake whitefish (*Coregonus clupeaformis*) (Mitchell and Seymour, 2003; Eme et al., 2015).

There is evidence that embryonic temperature has impacts on amphibian physiology beyond hatching. Such alterations in performance due to previous life history or conditions have been referred to as “carry-over effects” (O’Connor et al., 2014; Yagi and Green, 2018). Amphibians have shown carry-over/persistent effects from the thermal conditions of embryonic and/or larval development. For example, over test temperature ranges of 5°C–35°C, pre-metamorphic *Pseudacris regilla* (previously *Hyla regilla*) reared at 15°C as embryos swam faster and had higher myofibrillar ATPase activity than larvae reared at 25°C (Watkins, 2000). Similarly, sprint speed of larval *Bombina orientalis* was higher when larvae developed at relatively cooler temperatures (Parichy and Kaplan, 1995). *Rana sylvatica* that developed at 21°C as embryos compared to 15°C, swam slower when both developed at 21°C as larvae. However, embryonic development at 21°C and larval development at 18°C resulted in similar swimming speed compared to embryonic development at 15°C and larval development at 21°C, indicative of temperature exposure interactions across developmental time points (Watkins and Vraspir, 2005). Embryonic and larval acclimation temperatures showed interactive effects on swimming performance curves of *Limnodynastes peronii* larvae, with larvae incubated and acclimated at 25°C performing best in terms of breadth and peak of burst swimming speed when tested between 10 to 30°C (Seebacher and Grigaltchik, 2014). In contrast, *L. peronii* larvae incubated as embryos at 15°C did not show differences in larval $\dot{\text{V}}$O_2_ compared to those incubated at 25°C, despite higher $\dot{\text{V}}$O_2_ following larval acclimation to 15°C (Seebacher and Grigaltchik, 2014). Collectively, these studies suggest that within a range of acclimation temperatures, which may vary from species to species, relatively cooler or warmer temperatures during development of poikilothermic ectotherms may confer lower or higher performance during the larval stage, depending on the variable measured, supporting for example either the “warmer is better” or “colder is better” hypothesis of phenotypic plasticity (Huey et al., 1999). Furthermore, benefits of relatively cooler temperatures on performance are often reduced or absent at higher test temperatures (Wilson and Franklin, 1999; Watkins, 2000; Wilson et al., 2000; Seebacher and Grigaltchik, 2014), supporting the “beneficial acclimation hypothesis” that development at one temperature may be beneficial for performance at that temperature, but not at others (Huey et al., 1999). These previous studies examine function at one life history stage (e.g., larval stage) following embryonic and/or larval development under different temperatures. However, a deeper understanding of how temperature shapes developing phenotypes requires assessment of physiological function at *both* embryonic and larval life history stages.

The Baja California chorus frog (*Pseudacris hypochondriaca*) experiences a variable thermal environment across its life history. The chorus frog inhabits a wide range of environments throughout its latitudinal range (∼1400 km) from Santa Barbara County, California to Baja California, Mexico (Recuero et al., 2006), including deserts, grasslands, mountains and forests, that differ fundamentally in seasonal temperatures and elevation (∼3000 m) (Jameson et al., 1970; Schaub and Larsen, 1978). *P. hypochondriaca* reproduces in a variety of water bodies, some of which are temporary, and males initiate mating calls at a wide range of water temperatures, between 4 and 20°C (Brattstrom and Warren, 1955; Cunningham and Mullally, 1956), though it is likely they also call at even higher temperatures (Mueller, unpublished data). Previous observations indicated embryos develop successfully between 8 and 29°C, and larvae between 0 and 33°C (Brattstrom and Warren, 1955; Brown, 1975). Adult chorus frogs also appear to tolerate a wide range of temperatures (Cunningham and Mullally, 1956), but how developmental temperature may shape the physiological phenotype of this eurythermal amphibian is unknown.

**FIGURE 1:**
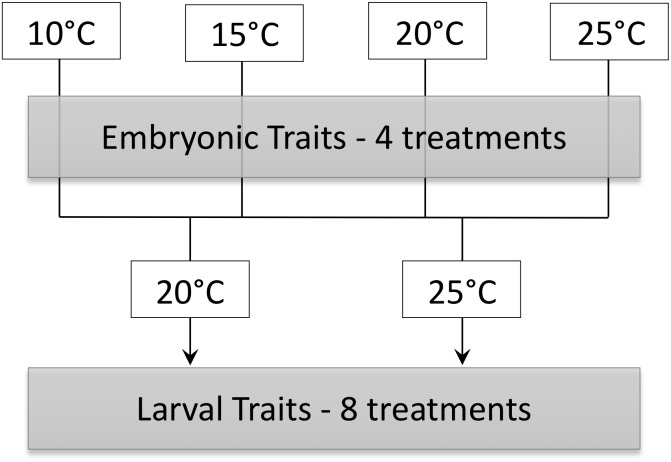
Schematic of experimental design in which embryos incubated at 10, 15, 20, and 25°C were acclimated to either 20 and 25°C as larvae.

The goal of this study was to determine immediate and possibly persistent effects of temperature on embryonic and larval *P. hypochondriaca*. Embryos were incubated in constant temperatures of 10, 15, 20, or 25°C until hatching to determine the immediate influence of temperature on survival, development rate, growth and energy use of embryos. We hypothesized that embryonic survival would be high across temperatures, similar to a previous study that did not see a decrease in survival until approximately 30°C (Brown, 1975). We also predicted that embryonic development rate would increase as temperature increased, and that hatchlings would be smaller at higher temperatures with greater residual yolk. We hypothesized that embryonic mass-specific oxygen consumption rate ($\dot{\text{V}}$O_2_) would remain constant or decrease throughout development prior to a marked increase for hatched animals, similar to previous data for embryonic and larval fish (Rombough, 1994; Eme et al., 2015). We also hypothesized that $\dot{\text{V}}$O_2_ would be greater at higher temperatures in later embryonic stages due to temperature dependent effects observed in ectotherm metabolism. Larvae from each embryonic temperature treatment (10, 15, 20, or 25°C) were acclimated to 20 or 25°C to assess the possibility that effects of embryonic environment carried-over into larval phenotypes (Figure 1). We predicted that embryonic temperature would have persistent effects on larval aerobic metabolism, with larvae incubated as embryos in colder temperatures showing higher mass-specific $\dot{\text{V}}$O_2_ at both larval acclimation temperatures, a “colder is better” result similar to a previous study showing such trends for $\dot{\text{V}}$O_2_ and aerobic enzyme activities (Huey et al., 1999; Seebacher and Grigaltchik, 2014). We also predicted that incubation at warmer temperature as embryos would confer greater thermal tolerance for larvae at the same acclimation temperature, a “warmer is better” result similar to a previous study that showed similar trends for swimming speed (Huey et al., 1999; Seebacher and Grigaltchik, 2014). Lastly, we hypothesized that acclimation to 25°C would result in lower mass-specific $\dot{\text{V}}$O_2_ and greater thermal tolerance of larvae compared to acclimation to 20°C.

## Materials and Methods

### Field Collection and Embryo Incubation

Eggs were collected from ponds on the California State University San Marcos campus (33°07′58.4″N/117°09′38.0″W, 200 m elevation) during January–February 2017 and January–March 2018. Eggs were collected during mid-late gastrulation (Gosner (1960) stages 8–9), indicating embryos were likely laid the night before and thus were within 24 h of fertilization. Eggs were cleaned of debris in water in the lab under a microscope (Zoom Stereo Trinocular Microscope, Amscope, Irvine, CA, United States). Eggs were collected, cleaned and incubated in 230 ml plastic Ziploc^®^containers with identically sourced water, tap water that had been aged by bubbling with air for 48 h before use. Eggs were initially collected and cleaned with aged water that was identical in temperature to field water (13–16°C). Clutches were separated and distributed between four temperatures: 10, 15, 20, and 25°C. The 10°C and 15°C embryos were incubated in wine coolers (Newair AWC-330E, Huntington Beach, CA, United States), 20°C embryos were incubated in a Thermo Scientific Precision Incubator (Marietta, OH, United States), and 25°C embryos were incubated in an environmental growth chamber (Chagrin Falls, Ohio, United States). A Thermochron iButton^®^data logger (Model DS1922L, iButtonLink, Whitewater, WI, United States) recorded temperature every 30 min in a separate container with the same volume of water as the embryo containers. Temperature was recorded every 30 min, and average daily temperatures were used to generate a grand mean for each temperature: 10°C = 10.0 ± 0.1°C, 15°C = 15.0 ± 0.1°C, 20°C = 19.9 ± 0.1°C, 25°C = 25.1 ± 0.1°C. Clutches were observed for mortality and first hatching, and the number of hatchlings was recorded each day until all embryos had hatched. Survival at 50% hatch, average time to 50% hatch, and hatch window duration (days from initial to final hatchling) were determined across clutches (10°C = 6 clutches, 15°C = 13 clutches, 20°C = 10 clutches, 25°C = 9 clutches).

### Embryo Oxygen Consumption Rate ($\dot{\text{V}}$O_2_)

Oxygen consumption rate ($\dot{\text{V}}$O_2_) was measured at Gosner embryonic stages 16, 18, 20, and 22 (hatch) in independent embryos at the temperature in which embryos were incubated (*n* = 8 per stage for each treatment). $\dot{\text{V}}$O_2_ was determined from the decrease in partial pressure of oxygen (PO_2_) in a closed respirometry system. Individual embryos/hatchlings were placed in respiratory chambers (volume: 0.94–1.17 ml) containing a 5 mm O_2_ sensor spot (Loligo System, Tjele, Denmark). Four chambers were housed in a recirculating water bath thermostated to the target temperature by a recirculating heater/chiller (Thermo Fisher Scientific, IsotempTM 6200, Waltham, MA, United States). The sensor spot in each chamber was read by a fiber optic cable (PreSens Precision Sensing, GmbH, Regensbury, Germany) connected to a Witrox 4 oxygen meter (Loligo Systems, Tjele, Denmark) with a computer running Autoresp^TM^ software (Loligo Systems, Tjele, Denmark). Oxygen levels of each chamber was read every 5 min for approximately 120 min. The first ∼40 min was excluded from $\dot{\text{V}}$O_2_ calculation to account for handling and allow embryos to reach a steady state of oxygen consumption. The lowest recorded oxygen levels in chambers were 12 kPa, and $\dot{\text{V}}$O_2_ was always stable (linear) over the 120 min measurement period, indicating O_2_ levels were above those likely to adversely affect $\dot{\text{V}}$O_2_. $\dot{\text{V}}$O_2_ (μl h^-1^) was calculated using the following equation:

$$\dot{V}{\text{O}}_{2}=\left(\frac{P{\text{O}}_{\text{2(t2)}}-{\text{PO}}_{\text{2(t1)}}}{t_{2}-t_{1}}\right)*\beta *\text{V}$$

where *PO_2(t2)_–PO_2(t1)_* is the decrease in PO_2_ (kPa) in the chamber over the time period (*t_2_–t_1_*, h), *β* is the capacitance of the water at the relevant temperature (μl O_2_ μl^-1^ kPa^-1^), and V is the chamber volume minus the embryo volume (μl, estimated from mass). Each chamber was calibrated with a fresh anoxic solution of 10 mg sodium sulfite in 1 ml of water (0%) and air equilibrated water (100%). All $\dot{\text{V}}$O_2_ values were corrected for $\dot{\text{V}}$O_2_ of the empty chamber to account for any microbial oxygen consumption. Mass-specific $\dot{\text{V}}$O_2_ (μl h^-1^ mg^-1^) was calculated by dividing $\dot{\text{V}}$O_2_ by dry yolk-free mass.

### Embryo Mass

Individuals used for $\dot{\text{V}}$O_2_ determination and other embryos selected at embryonic stages 16, 18, 20, and 22 (hatch) were placed in 4% paraformaldehyde (PFA) in phosphate buffered saline (PBS; pH = 7.4) in microcentrifuge tubes. After fixation in 4% PFA for 36 ± 1 d, embryos/hatchlings were removed from their chorion (if not already so) and photographed under a dissecting microscope (10-15X, Zoom Stereo Trinocular Microscope, Amscope, Irvine, CA, United States) with a USB 3.0 digital camera (Amscope, Irvine, CA, United States). The yolk was then carefully dissected away from the embryo, and the embryo and yolk weighed to ± 0.01 mg (XA105DU, Mettler-Toledo, Columbus, OH, United States). The embryo and yolk were dried at 65°C for 24 h (model 10 oven, Quincy Lab, Inc., Chicago, IL, United States) and reweighed. The stage of each embryo was confirmed from photographs.

### Larval Incubation

A subset of embryos were allowed to hatch and were maintained at each embryo temperature treatment until 5–7 d after first feeding. Larvae in each temperature were then randomly separated into two groups and acclimated to 20 or 25°C. Temperature of the larvae was increased at a rate of 1–2.5°C day^-1^. This created 8 larval temperature treatments (Figure 1). Larvae were maintained at each temperature in aged tap water in 2 L rectangular plastic containers at a density of 5–8 larvae L^-1^ and a 12 h: 12 h light: dark regime. Twice weekly, containers were cleaned of waste, 30% of the water replaced with fresh aged water, and larvae fed boiled lettuce *ad libitum*.

### Larval Routine Oxygen Consumption Rate and Critical Thermal Maximum

Larval development was monitored until Gosner (1960) stages 32–36, which represents hindlimb toe differentiation. Larvae were acclimated to 20 or 25°C for a minimum of 2 weeks prior to reaching these stages. The average acclimation period for each temperature treatment prior to experimentation was as follows: 10–20°C: 52 ± 3 d, 15–20°C: 46 ± 2 d, 20–20°C: 40 ± 3 d, 25–20°C: 31 ± 2 d, 10–25°C: 53 ± 1 d, 15–25°C: 34 ± 3 d, 20–25°C: 27 ± 2 d, and 25–25°C: 32 ± 3 d.

Once the larva had reached the representative stage, routine $\dot{\text{V}}$O_2_ was measured at the acclimation temperature using intermittent respirometry (*n* = 6–16 per treatment). Individual larvae were placed in ∼8.2 ml respirometry chambers containing a 5 mm O_2_ sensor spot (Loligo System, Tjele, Denmark) and read as described above for embryonic $\dot{\text{V}}$O_2_. An intermittent cycle of 60 s flush, 240 s wait and 300 s measure periods was controlled by a Daq-M and Autoresp^TM^software (Loligo Systems, Tjele, Denmark). This intermittent cycle resulted in PO_2_ of the chambers never falling below 17 kPa. Trials were performed for a minimum of 120 min, and the first ∼40 min was excluded from $\dot{\text{V}}$O_2_ calculation to account for handling and allow larvae to reach a steady state of oxygen consumption. Each chamber was calibrated with a fresh anoxic solution of 10 mg sodium sulfite in 1 ml of water (0%) and air equilibrated water (100%). It was not possible to keep larvae completely stationary during trials, so measurements represent routine metabolic rate (i.e., $\dot{\text{V}}$O_2_ that represents O_2_ consumed during relatively low levels of activity). All $\dot{\text{V}}$O_2_ values were corrected for $\dot{\text{V}}$O_2_ of empty chambers to account for any microbial oxygen consumption. Following $\dot{\text{V}}$O_2_ determination, larvae were euthanized in MS-222, staged, weighed and fixed in 4% PFA in PBS. After fixation for ∼30 days, the gut was dissected from the larvae and wet and dry masses determined as described above for embryos.

Critical thermal maximum (CTMax) was determined for a separate subset of larvae (*n* = 5–14 per treatment) at the same developmental stages as $\dot{\text{V}}$O_2_ measurement. For each CTMax trial, individual larvae were placed in 250 ml tri-corner plastic beakers (Globe Scientific, Inc., Paramus, NJ, United States) filled with aged tap water at the appropriate temperature. Beakers were suspended in a polystyrene frame within a 20 L polystyrene-insulated plastic water bath that was heated continuously during the trial by two 150W aquarium heaters (EHEIM GmbH) and bath water circulated with an aquarium pump (15 L min^-1^, Lifeguard Aquatics, Cerritos, CA, United States). All individual test chambers were provided with moderate aeration to prevent thermal stratification. Beaker water temperatures were measured with a VWR^®^Traceable^®^Ultra waterproof thermometer (± 0.1°C) (VWR, Radnor, PA, United States). Temperatures were increased at 0.33 ± 0.01°C min^-1^. Temperature increase continued until larvae exhibited loss of equilibrium (LOE), defined as the inability to respond to mechanical stimulation and dorso-ventral orientation, and thus failure to show an escape response (Cupp, 1980; Sherman and Levitis, 2003). As tadpoles reached LOE, beaker temperature was recorded, and the temperature corrected based on a calibrated VWR^®^Traceable^®^Digital Thermometer (precision ± 0.0001°C; accuracy ± 0.05°C; Avantor, VWR, Radnor, PA, United States). Following LOE, larvae were immediately removed from the CTMax chamber and returned to their acclimation temperature. Larvae were allowed to recover for ≥1 h, after which they were euthanized in MS-222, dried and weighed to 0.01 mg. 90% of larvae survived for ≥1 h following trials, and only CTMax values for those that survived were included in analyses.

### Statistical Analyses

Data were tested for normality and homogeneity of variances using the Shapiro-Wilk and O’Brien test, respectively, and appropriate parametric or non-parametric tests used. Non-parametric analyses were used as no transformations ensured non-parametric data met parametric assumptions. Time to 50% hatch, hatch duration, survival at 50% hatch were compared across embryonic incubation temperature using a Kruskal-Wallis test with *post hoc* Steel-Dwass comparisons. Wet mass, dry mass, and water content of embryos and yolk across developmental stages and temperature were examined using a Friedman’s non-parametric 2-way ANOVA with developmental stage, temperature, and the interaction between developmental stage and temperature as the effects. Mass-specific $\dot{\text{V}}$O_2_ across developmental stages and temperature were examined with a 2-way ANOVA with developmental stage, temperature, and the interaction between developmental stage and temperature as the effects. *Post hoc* Tukey HSD comparisons were used when effects were significant.

Larval mass-specific $\dot{\text{V}}$O_2_ were compared across temperature treatments using a two-way ANOVA with embryonic temperature, larval acclimation temperature, and the interaction between embryonic and larval temperatures as the effects. Tukey HSD comparisons were used when effects were significant. Larval CTMax was compared across temperature treatments using a 2-way ANCOVA with embryonic temperature and larval acclimation temperature as the main effects and dry mass as a covariate. Data are presented as mean ± SEM, and differences were accepted as statistically significant at α = 0.05.

## Results

### Embryonic Development, Growth and Oxygen Consumption

**FIGURE 2:**
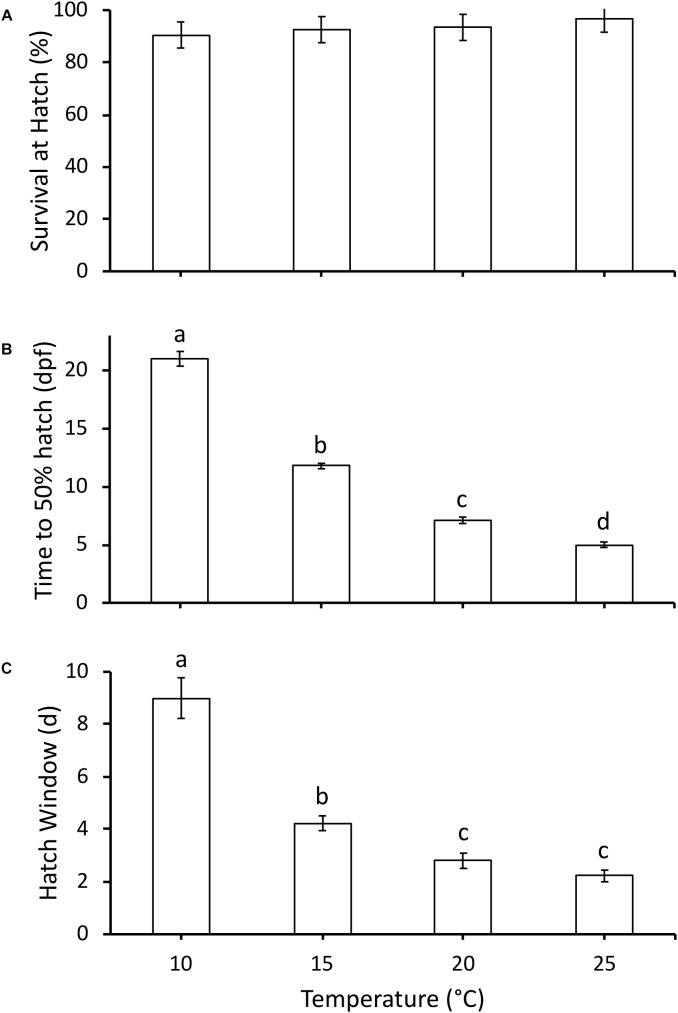
**(A)** Survival at hatch (%), **(B)** time to 50% hatch (days post-fertilization, dpf), and **(C)** hatch window (days from first to last hatch) of hatchlings incubated at different temperatures. Data presented as mean ± SEM, n for each temperature as follows: 10°C = 6, 15°C = 13, 20°C = 10, 25°C = 9. Different letters indicate significant differences between temperatures based on Kruskal-Wallis test and Steel-Dwass *post hoc* comparisons.

All embryos hatched at Gosner (1960) stage 22. Survival (%) was high throughout embryonic development and not affected by temperature, with 92–97% survival at 50% hatch across all temperatures (χ^2^_3_ = 3.8, *P* = 0.28, Figure 2A). As temperature increased, time to 50% hatch significantly decreased from 21 days post-fertilization (dpf) at 10°C to 5 dpf at 25°C (χ^2^_3_ = 34.2, *P* < 0.0001, Figure 2B). Q_10_ for development rate was 2.8 for 10–15°C, 3.1 for 15–20°C and 2.0 for 20–25°C, with an overall Q_10_ of 2.5 for 10–25°C. Hatch duration (d) also significantly decreased with an increase in incubation temperature, but did not significantly differ between 20 and 25°C (χ^2^_3_ = 27.5, *P* < 0.0001, Figure 2C).

**FIGURE 3:**
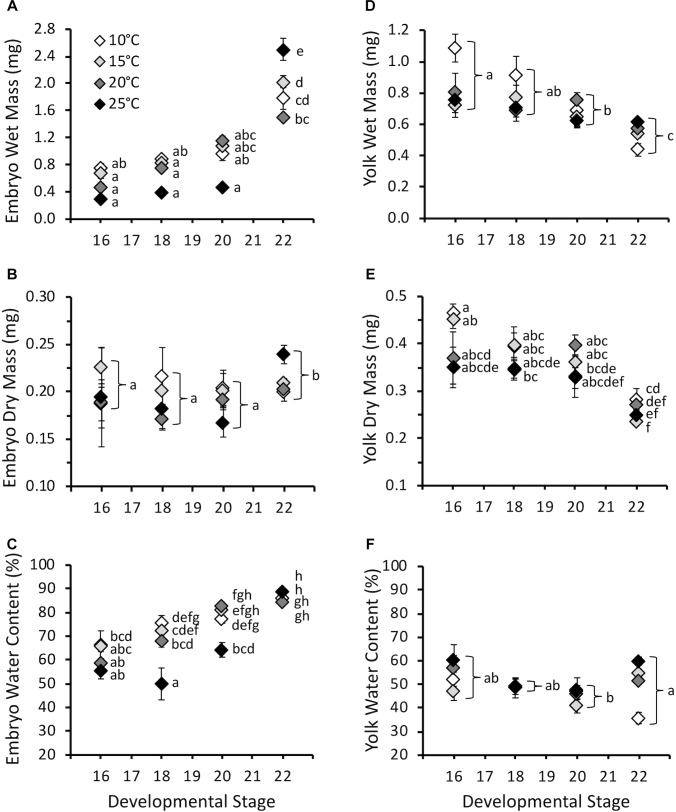
**(A)** Embryo wet mass (mg), **(B)** embryo dry mass (mg), **(C)** embryo water content (%), **(D)** yolk wet mass (mg), **(E)** yolk dry mass (mg), and **(F)** yolk water content (%) throughout embryonic development at different incubation temperatures. Developmental stages based on Gosner (1960). Data presented as mean ± SEM, *n* = 5–11 for stage 16, 7–20 for stage 18, 8–16 for stage 20, 39–121 for stage 22. Letters on **A**, **C,** and **E** indicate significant differences based on interaction between development stage and temperature (two-way ANOVA, Tukey *post hoc* comparisons). Letters on **B**, **D,** and **F** indicate significant differences between stages for all temperatures combined (two-way ANOVA, Tukey *post hoc* comparisons).

Embryo wet mass increased with developmental stage (*F*_3,387_ = 87.7, *P* < 0.0001), and was influenced by the interaction of stage and temperature (*F*_9,387_ = 5.5, *P* < 0.0001), but not temperature (*F*_3,387_ = 1.4, *P* = 0.26) (Figure 3A). Embryo wet mass was similar across temperatures at stage 16, 18, and 20. At stage 22 (hatching) embryo wet mass was higher at 25°C compared to other temperatures, and embryo wet mass at 15°C was higher than at 20°C. Embryo dry mass at hatching for all temperatures combined was significantly higher compared to earlier stages (*F*_3,380_ = 2.7, *P* = 0.04), but was not affected by temperature (*F*_3,380_ = 1.4, *P* = 0.24), or the interaction of stage and temperature (*F*_9,380_ = 1.7, *P* = 0.08) (Figure 3B). The majority of the increase in wet mass during development was due to an increase in water content, which increased with stage (*F*_3,380_ = 113.8, *P* < 0.0001), and was also significantly affected by temperature (*F*_3,380_ = 14.9, *P* < 0.0001) and the interaction of stage and temperature (*F*_9,380_ = 6.8, *P* < 0.0001) (Figure 3C). The significant interaction occurred due to lower embryo water content at stage 18 for 25°C embryos compared to other temperatures, and lower water content at stage 20 for 10 and 15°C embryos. Differences were absent by stage 22.

Yolk wet mass decreased with stage (*F*_3,384_ = 20.2, *P* < 0.0001) and was influenced by temperature (*F*_3,384_ = 4.7, *P* < 0.01), but not the interaction of stage and temperature (*F*_9,384_ = 1.8, *P* = 0.07) (Figure 3D). For all stages combined yolk wet mass was higher at 10°C compared to other temperatures. Yolk dry mass decreased with stage (*F*_3,379_ = 46.1, *P* < 0.0001), and was affected by temperature (*F*_3,379_ = 4.0, *P* < 0.01) and the interaction of stage and temperature (*F*_3,379_ = 3.3, *P* < 0.001) (Figure 3E). The significant interaction was due to higher yolk dry mass at hatching for 10°C embryos compared to 15 and 25°C. Yolk water content was lowest at stage 20 compared to stage 16 and 22 for all temperatures combined (*F*_3,379_ = 5.0, *P* < 0.01), but was not affected by temperature (*F*_3,379_ = 2.7, *P* = 0.09) or the interaction of stage and temperature (*F*_9,379_ = 1.5, *P* = 0.14) (Figure 3F).

**FIGURE 4:**
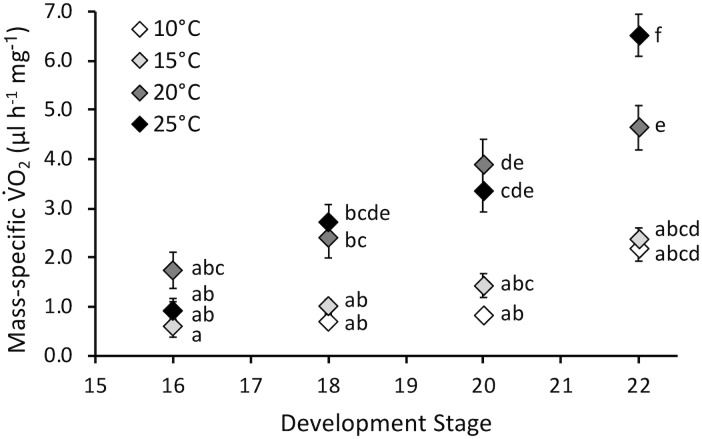
Mass-specific oxygen consumption rate ($\dot{\text{V}}$O_2_, μl h^-1^ mg^-1^) of embryos incubated at different temperatures. Developmental stages based on Gosner (1960). Data presented as mean ± SEM, *n* = 8 for all data points. Letters indicate significant differences based on interaction between development stage and temperature (two-way ANOVA, Tukey *post hoc* comparisons).

Embryonic mass-specific $\dot{\text{V}}$O_2_ was influenced by stage (*F*_3,130_ = 38.7, *P* < 0.0001) and temperature (*F*_3,130_ = 35.5, *P* < 0.0001) and the interaction of stage and temperature (*F*_9,130_ = 4.4, *P* < 0.0001) (Figure 4). The interaction occurred because mass-specific $\dot{\text{V}}$O_2_ did not significantly change across stages for embryos incubated at 10 and 15°C, but did increase across development at 20 and 25°C. Mass-specific $\dot{\text{V}}$O_2_ was not affected by temperature at stage 16. However, as development progressed mass-specific $\dot{\text{V}}$O_2_ diverged, and it was significantly different for 10 and 15°C embryos compared to 20 and 25°C at stages 18 and 20. At hatch (stage 22) mass-specific $\dot{\text{V}}$O_2_ increased with temperature, except for statistically similar values at 10 and 15°C. Q_10_ values for mass-specific $\dot{\text{V}}$O_2_ at hatching were 1.2 for 10–15°C, 3.8 for 15–20°C and 2.0 for 20–25°C, with an overall Q_10_ of 2.1 for 10–25°C.

### Larval Oxygen Consumption Rate and Critical Thermal Maximum

**FIGURE 5:**
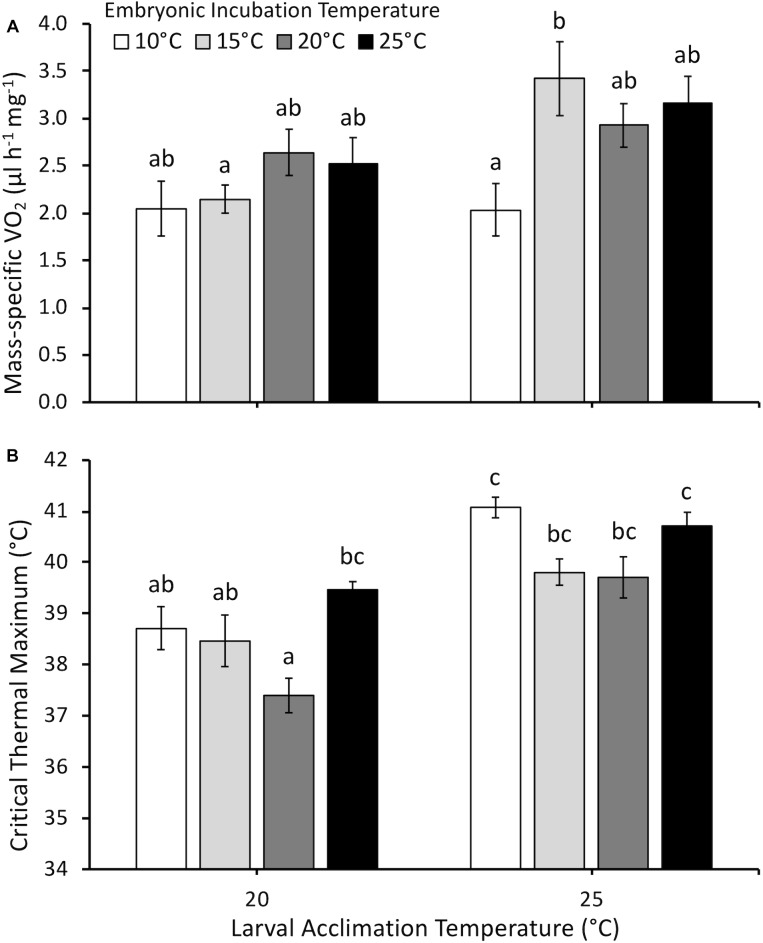
**(A)** Mass-specific oxygen consumption rate ($\dot{\text{V}}$O_2_, μl h^-1^ mg^-1^) and **(B)** critical thermal maximum (°C) of larvae acclimated to either 20 or 25°C following embryonic incubation at different temperatures. Larvae measured at Gosner (1960) stage 32–36 (hind limb toe differentiation). Data presented as mean ± SEM, *n* = 6–16 for $\dot{\text{V}}$O_2_, *n* = 5–14 for CTMax. Different letters indicate significant differences across all embryonic incubation and larval acclimation treatments (two-way ANOVA, Tukey *post hoc* comparisons).

Larval mass-specific $\dot{\text{V}}$O_2_ was influenced by embryonic temperature (*F*_3,75_ = 2.5, *P* = 0.035), larval acclimation temperature (*F*_1,75_ = 7.9, *P* < 0.01), and the interaction between embryonic and larval temperatures (*F*_3,75_ = 2.8, *P* = 0.044) (Figure 5A). Embryonic temperature influenced mass-specific $\dot{\text{V}}$O_2_ only at the larval acclimation temperature of 25°C. Following acclimation to 25°C, larvae incubated at 20°C as embryos demonstrated higher mass-specific $\dot{\text{V}}$O_2_ than larvae incubated at 10°C as embryos.

Critical thermal maximum was significantly affected by embryonic temperature (*F*_3,58_ = 7.0, *P* < 0.001), larval acclimation temperature (*F*_1,58_ = 44.0, *P* < 0.0001), the interaction between embryonic and larval temperatures (*F*_3,58_ = 3.5, *P* = 0.028), but not dry mass (*F*_1,58_ = 2.9, *P* = 0.1). Mean CTMax was 1.8°C higher at an acclimation temperature of 25°C compared to 20°C (Figure 5B). However, embryonic temperature had persistent effects in certain cases. At the tadpole acclimation temperature of 20°C, larvae incubated at 20°C as embryos had lower CTMax than larvae incubated at 25°C. In contrast, CTMax was similar across all embryonic temperature treatments at 25°C acclimation.

## Discussion

One of the most dynamic sources of physiological phenotypic plasticity is environmental temperature early in development, and predicting persistent physiological consequences for organisms across taxa is an area of significant current research interest (Kim et al., 2017; Noble et al., 2018; Ruthsatz et al., 2018; Warner et al., 2018). We explored this central idea in comparative physiology using *P. hypochondriaca*, an amphibian that experiences a broad range of temperatures throughout its life history. We demonstrated that temperature during embryonic development had both immediate embryonic effects as well as carry-over larval effects that influenced physiological function. Increased temperature during embryonic development did not reduce survival or mass, despite an increased energy demand that supported faster development. Acclimation to higher temperature during larval development generally caused increased thermal tolerance, whereas aerobic metabolism showed no change or a modest increase with higher larval acclimation temperature. Embryonic incubation temperature had certain persistent effects. Namely, embryonic temperature of 10°C resulted in reduced $\dot{\text{V}}$O_2_ at a larval acclimation temperature of 25°C, and embryonic development at 25°C resulted in high thermal tolerance in 20°C acclimated larvae. Therefore, in a thermally robust species such as *P. hypochondriaca* the developmental environment has long-term impacts on phenotype. This is particularly important in relation to temperature, as it may provide valuable insight into species responses to a changing climate.

### Immediate Effects of Temperature During Embryonic Development

The embryonic responses to temperature in *P. hypochondriaca* demonstrated broad thermal tolerance of the species. Development until hatching was highly successful across the temperature range (10 – 25°C) studied (Figure 2A), matching previous studies that indicate successful embryonic development at temperatures up to 29°C (Brown, 1975). However, it was somewhat surprising that survival remained so high (97%) at 25°C. This temperature has been rarely recorded in the field location from which the eggs were collected (Mueller, unpublished data), yet the embryos show no apparent adverse effects. While survival was consistent across treatments, temperature did drive a step-wise increase in embryonic development rate, as reflected in a decrease in time to 50% hatch (Figure 2B) and a reduction in hatch window (Figure 2C). Increased development rate with temperature was an expected result, and has been observed in many amphibians (McLaren and Cooley, 1972; Kuramoto, 1975; Bradford, 1990; Seymour et al., 1991; Mitchell and Seymour, 2000). The Q_10_ for development rate was 2.5 across the temperature range studied, which is in the lower range of Q_10_ values (2.0 to 4.5) measured in other amphibians across the same temperature range (Kuramoto, 1975). A relatively low Q_10_ indicates that temperature has less of an effect on development rate compared to other species, which provides additional support for the broad thermal tolerance of *P. hypochondriaca*. Together, the survival and development rate results highlight the eurythermal nature of the embryos and suggests that *P. hypochondriaca* is likely to be of least concern even if air temperatures increase by 1.6–4.4°C, and water temperatures increase by 0.5–2.5°C by 2099, as predicted by different climate models for southern California (Cayan et al., 2008; van Vliet et al., 2013).

Despite the effect of temperature on development rate, temperature had limited influence on masses throughout embryonic development (Figure 3). This result does not support our hypothesis that warmer embryos would be smaller at hatch, as has been observed in other amphibian and fish species (Rombough, 1988; Kamler et al., 1998; Mueller et al., 2015b; Eme et al., 2018). However, other species have also shown no change in body mass with temperature, so this is not unique to *P. hypochondriaca* (Watkins and Vraspir, 2005; Mueller et al., 2011). While embryo wet mass increased throughout embryonic development, this was largely due to an increase in water content, and there was limited increase in dry mass only at hatching. In contrast, both wet and dry yolk mass decreased, while yolk water content was relatively stable. The lack of substantial somatic growth prior to hatch has been observed in other amphibians (Mitchell, 2001; Mueller et al., 2012). The limited growth at these stages is likely to limit the influence that temperature may have in how energy is allocated to mass. However, we did not observe differences in larvae mass when larval $\dot{\text{V}}$O_2_ and CTMax were measured, and therefore the limited temperature effect on mass appears to persist throughout larval development at the temperatures examined.

Contrary to our hypothesis, mass-specific $\dot{\text{V}}$O_2_ increased throughout embryonic development at higher temperatures, and this matched increased development rate (Figure 4). Overall Q_10_ values for development rate (2.5) and mass-specific $\dot{\text{V}}$O_2_ (2.1) were similar, indicating comparable temperature sensitivity for these two important developmental rates. Thus, immediate effects of temperature on embryonic development, namely faster development at increased temperature, is largely correlated with increased energy use. There does not appear to be a decrease in energy efficiency with development at higher temperatures. These findings further indicated that temperatures up to 25°C appeared to have no significant immediate negative impact on *P. hypochondriaca* embryos. Examining energy allocation during development to the processes such as development, growth and maintenance, which can be modeled with dynamic energy budget theory (Mueller et al., 2012), would be a fruitful area of future study in this species.

### Interactive Effects of Embryonic Temperature and Larval Acclimation Temperature

Our investigation of larval function revealed the effects of larval acclimation temperature and persistent effects of embryonic temperature. We examined both mass-specific $\dot{\text{V}}$O_2_ and CTMax during hindlimb toe differentiation [Gosner (1960) stages 32–36] across all temperature treatments. By nature of temperature influencing development rate, the period of acclimation prior to reaching these stages was shorter at higher temperatures, and there is a possibility this may have influenced the results observed. However, *P. hypochondriaca* shows variation in larval development duration within temperatures (Mueller, unpublished data), and every animal measured was acclimated to its larval temperature for at least 2 weeks. Instead, we hypothesized that the developmental status of an individual is more likely to have an impact on function, and thus we performed our measurements within a narrow range of developmental stages, which resulted in comparable larval sizes across treatments.

Persistent effects of the embryonic environment on larval mass-specific $\dot{\text{V}}$O_2_ or CTMax depended upon larval acclimation temperature, with both measurements supporting a “warmer is better” effect of embryonic incubation temperature (Huey et al., 1999). Larval mass-specific $\dot{\text{V}}$O_2_ measured following acclimation to 20°C showed no significant persistent effects of embryonic temperature (Figure 5A). However, at the acclimation temperature of 25°C, we observed that larvae incubated at 10°C as embryos had reduced mass-specific $\dot{\text{V}}$O_2_ compared to larvae incubated at 15°C. This result is in contrast to our hypothesis that embryonic incubation at colder temperatures would cause higher mass-specific $\dot{\text{V}}$O_2_ at later developmental stages (i.e., “colder is better”). Our data showed that larval mass-specific $\dot{\text{V}}$O_2_ either increases or does not change at the higher larval acclimation temperature of 25°C compared to 20°C. Seebacher and Grigaltchik (2014) showed that *Limnodynastes peronii* larvae displayed higher $\dot{\text{V}}$O_2_ when acclimated to 15°C compared to 25°C, particularly when measured at 20, 25 and 30°C (Seebacher and Grigaltchik, 2014). The present studies’ acclimation temperatures were only 5°C apart compared to 10°C used by Seebacher and Grigaltchik (2014), and the general trend in *P. hypochondriaca* was for higher acclimation temperature to result in higher $\dot{\text{V}}$O_2_. It is possible that different larval acclimation periods (∼2–8 weeks for this study, 6 weeks for *L. peronii*) can result in different observations for the effect of embryonic incubation temperature on $\dot{\text{V}}$O_2_.

It is also possible that any persistent effects of embryonic incubation temperatures are species-specific and/or trait-specific. Only the present study and Seebacher and Grigaltchik (2014) measured multiple whole animal performance or enzymatic data, and both studies show separate dependent variables that support either cooler (Seebacher and Grigaltchik, 2014 $\dot{\text{V}}$O_2_ and aerobic enzyme assays) or warmer (Seebacher and Grigaltchik, 2014 anaerobic enzyme assays; and present study, $\dot{\text{V}}$O_2_) incubation temperatures conferring a higher energy use phenotype for larvae. Seebacher and Grigaltchik (2014) also showed persistently higher values for a performance trait (swimming speed) following warmer embryonic incubation, similar to our finding for $\dot{\text{V}}$O_2_ and CTMax data. However, *R. sylvatica* that developed at cooler temperatures swam faster than warmer incubated embryos, when measured as larvae, suggesting cooler temperatures are better for larval performance measurements (Watkins and Vraspir, 2005).

Similar to mass-specific $\dot{\text{V}}$O_2_, interactive effects between embryonic temperature and larval acclimation temperature resulted in changes in larval thermal tolerance. Most treatments in the present study showed that acclimation to a higher temperature conferred greater thermal tolerance in *P. hypochondriaca* larvae (Figure 5B). At the larval acclimation temperature of 20°C, larvae incubated at 20°C as embryos had the lowest CTMax, significantly lower than larvae incubated at 25°C as embryos. In fact, CTMax of larvae incubated at 25°C as embryos was relatively high compared to other treatments at this acclimation temperature, and was not significantly different from CTMax measured at 25°C. These larvae were the only treatment in which the change from embryonic to larval temperature involved a decrease in temperature rather than an increase or no change. The increased embryonic temperature appears to have conferred a higher thermal tolerance that was retained, even following larval acclimation to a colder temperature. Thus, this treatment supported our hypothesis that warmer embryonic temperatures would result in increased thermal tolerance of larvae. Future experiments could examine a decrease in larval compared to embryonic temperature to provide insight into effects on thermal tolerance.

Thermal tolerance of larval *P. hypochondriaca* is within the range measured in similar species, but was more responsive to acclimation compared to previous studies. Our CTMax values at an acclimation temperature of 20°C (38–39°C) were similar to *Pseudacris triseriata*, *R. sylvatica*, *R. temporaria*, and *Xenopus laevis*, but lower than values (41–43°C) observed in *Bufo americanus*, *B. woodhousei*, *B. marinus*, and *Gastrophryne carolinenus* at the same acclimation temperature (Cupp, 1980; Floyd, 1983; Sherman and Levitis, 2003; Enriquez-Urzelai et al., 2019). Thus, thermal tolerance of *P. hypochondriaca* larvae tends to match other species that breed during the winter months. Across embryonic treatments, a 5°C increase in acclimation temperature resulted in an average 1.8°C increase in CTMax (slope of change in temperature tolerance for every 1°C increase in acclimation temperature = 0.36). This is a greater gain of tolerance that observed in other species. For example, when measured at the same developmental stages, *R. temporaria* gains 0.8°C with a 7°C increase in acclimation temperature (slope = 0.11), while *R. catesbeiana* and *B. marinus* gain approximately 0.7 and 1°C, respectively, with a 10°C increase in acclimation temperature (slopes = 0.07 and 0.1, respectively) (Menke and Claussen, 1982; Floyd, 1983; Enriquez-Urzelai et al., 2019). Performing similar experiments in species that vary in their temperature ranges during development may help to fully understand the effects of acclimation and potential carry-over effects of temperature on thermal tolerance.

## Summary

This study confirmed the thermally tolerant nature of *P. hypochondriaca* during its embryonic and larval life stages, and we demonstrated that the embryonic environment can have persistent effects at the larval stage. We observed that embryonic temperature of 10°C resulted in reduced $\dot{\text{V}}$O_2_ at a larval acclimation temperature of 25°C, whereas CTMax was higher in larvae incubated at 25°C and acclimated to 20°C. Thus, under what conditions carry-over effects of developmental temperature emerge in the phenotype are likely to be trait-specific. It remains unclear what the implications of changes in energy use and thermal tolerance are for *P. hypochondriaca* beyond the larval stage. Future studies should examine phenotypes across the metamorphic transition to see if carry-over effects emerge even following significant morphological and physiological changes such as those occurring during amphibian metamorphosis. Higher larval temperatures generally produce smaller and younger metamorphs (see meta-analysis by Ruthsatz et al., 2018), but the functional consequences of this beyond metamorphosis is unclear; in our present study high embryonic incubation temperature did not affect hatchling size of these eurythermal frogs. Larval density has been shown to influence size and digestive morphology after metamorphosis (Bouchard et al., 2016; Yagi and Green, 2018). A recent study with *R. temporaria* suggests larval thermal tolerance may not persist beyond metamorphosis, and therefore water temperature exposure during larval development may not confer protective or deleterious effects on juveniles (Enriquez-Urzelai et al., 2019).

Future studies exploring physiology in multiple amphibian species are required to elucidate general patterns in phenotypic responses to developmental temperature. Our study partially supported a “warmer is better” effect of embryonic incubation temperature (similar or higher $\dot{\text{V}}$O_2_ and CTMax for larvae incubated in warmer embryonic conditions), whereas Seebacher and Grigaltchik (2014) showed support for the “colder is better” hypothesis (higher $\dot{\text{V}}$O_2_ and aerobic enzyme activities for larvae incubated in colder embryonic conditions (Huey et al., 1999; Seebacher and Grigaltchik, 2014). Moving forward, comparisons of stenothermal versus eurythermal species, and studies including amphibians that differ in their geographic ranges and temperatures they experience during development are needed to understand the factors that influence persistent phenotypic changes. Amphibians with restricted geographic distributions show increased stress responses compared to generalist species with larger distributions (Assis et al., 2018), and it is likely that stenothermal amphibians may also show stronger persistent phenotypic changes in response to temperature compared to eurythermal species such as *P. hypochondriaca*. Examining effects of developmental temperature across life history of numerous species will provide us with a more comprehensive understanding of how temperature shapes phenotypes, and this will be invaluable for predicting species responses to a changing climate.

## Data Availability

The datasets generated for this study are available on request to the corresponding author.

## Ethics Statement

This study was carried out in accordance with the recommendations of the Office for Laboratory Animal Welfare, the National Institutes of Health, and the California State University San Marcos Institutional Animal Care and Use Committee. The protocol was approved by the California State University San Marcos Institutional Animal Care and Use Committee.

## Author Contributions

CM conceived and designed the study, performed the experiments, and wrote the manuscript. JB, LK, and SM performed the experiments and collated the data. All authors approved final version of the manuscript.

## Conflict of Interest Statement

The authors declare that the research was conducted in the absence of any commercial or financial relationships that could be construed as a potential conflict of interest.
